# Association between ethnicity and obesity with high-density lipoprotein (HDL) function and subclass distribution

**DOI:** 10.1186/s12944-016-0257-9

**Published:** 2016-05-11

**Authors:** Nicholas J. Woudberg, Julia H. Goedecke, Dee Blackhurst, Miguel Frias, Richard James, Lionel H. Opie, Sandrine Lecour

**Affiliations:** Department of Medicine, Hatter Institute for Cardiovascular Research in Africa and South African Medical Research Council Inter-University Cape Heart Group, Faculty of Health Sciences, University of Cape Town, Chris Barnard Building, Anzio Road, Observatory, 7925 Cape Town, Western Cape South Africa; Non-Communicable Disease Research Unit, South African Medical Research Council, Cape Town, South Africa; Department of Human Biology, University of Cape Town, Cape Town, South Africa; Division of Chemical Pathology, Department of Pathology, University of Cape Town, Cape Town, South Africa; Department of Internal Medicine, Faculty of Medicine, University of Geneva, Geneva, Switzerland

**Keywords:** High-density lipoprotein, Ethnicity, Obesity, Cardiovascular risk

## Abstract

**Background:**

Obesity and low high-density lipoprotein-cholesterol (HDL-C) levels are associated with cardiovascular risk. Surprisingly, despite a greater prevalence of obesity and lower HDL concentrations than white women, black South African women are relatively protected against ischaemic heart disease.

**Methods:**

We investigated whether this apparent discrepancy may be related to different HDL function and subclass distribution in black and white, normal-weight and obese South African women (*n* = 40). HDL functionality was assessed by measuring paraoxonase (PON) activity, platelet activating factor acetylhydrolase (PAF-AH) activity, Oxygen Radical Absorbance Capacity (ORAC) and quantification of the expression of vascular cell adhesion molecule in endothelial cells. PON-1 and PAF-AH expression was determined in isolated HDL and serum using Western blotting. Levels of large, intermediate and small HDL subclasses were measured using the Lipoprint® system.

**Results:**

PON activity was lower in white compared to black women (0.49 ± 0.09 U/L vs 0.78 ± 0.10 U/L, p < 0.05), regardless of PON-1 protein levels. Obese black women had lower PAF-AH activity (9.34 ± 1.15 U/L vs 13.89 ± 1.21 U/L, p <0.05) and HDL-associated PAF-AH expression compared to obese white women. Compared to normal-weight women, obese women had lower large HDL, greater intermediate and small HDL; an effect that was more pronounced in white women than black women. There were no differences in antioxidant capacity or anti-inflammatory function across groups.

**Conclusions:**

Our data show that both obesity and ethnicity are associated with differences in HDL functionality, while obesity was associated with decreases in large HDL subclass distribution. Measuring HDL functionality and subclass may, therefore, be important factors to consider when assessing cardiovascular risk.

**Electronic supplementary material:**

The online version of this article (doi:10.1186/s12944-016-0257-9) contains supplementary material, which is available to authorized users.

## Background

Although the leading cause of death in Sub-Saharan Africa remains communicable diseases, the prevalence of ischemic heart disease is increasing and it is predicted to be the leading cause of death in low-income countries by 2030 [1–3]. The changes in standardized health care, progressive changes in socio-economic status and greater Westernization have raised the burden of preventable cardiovascular diseases (CVD) [4–6]. In addition, nearly 23 % of the worldwide burden of ischaemic heart disease can be attributed to obesity, the prevalence of which has doubled since 1980 [7]. Obesity is associated with insulin resistance, which may increase the risk of type II diabetes and dyslipidaemia, as evidenced by an increase in triglycerides and low-density lipoproteins cholesterol (LDL-C) and a decrease in high-density lipoproteins cholesterol (HDL-C) in these patients [8–11].

Ethnic differences in lipid profiles and CVD risk have been documented, attributed to, in part, genetic, socioeconomic and lifestyle differences [5, 12]. Black South African women and African Americans exhibit protective lipid profiles, characterised by low LDL-C, low triglyceride and low total cholesterol concentrations [13, 14]. In addition, cholesterol-attributable mortality is higher in South African white compared to black populations, with only 1.8 % mortality attributable to ‘sub-optimal’ cholesterol levels in black populations [15]. It was previously thought that a favourable lipid profile in black populations would also be characterised by higher HDL-C concentrations [16, 17]. However, recent studies conducted in black South African women highlighted a lower or equivalent level of HDL-C than their white counterparts [6, 12, 14, 18].

The Framingham Heart study suggests an inverse relationship between HDL-C levels and cardiovascular risk [19]. However, recent clinical trials aiming to reduce cardiovascular complications by raising HDL-C levels have shown disappointing results [20, 21]. Elucidation of the complexity of the HDL molecule, has led to a shift from measuring the quantity of HDL-C to assessing the composition, the distribution of individual HDL subclasses and HDL functionality to try to explain the relationship between HDL and cardiovascular risk [22, 23].

The risk of ischemic heart disease in African populations has largely been defined by classical risk factors, including the cholesterol component of HDL. Given the prevailing doubts about the cardiovascular value of HDL-C, the present study used blood collected from a population of black and white, obese and normal-weight South African women, to explore whether ethnicity and obesity may be associated with differences in HDL functionality and subclass distribution.

## Methods

### Subjects

The sample population consisted of 40 normal-weight (Body Mass Index [BMI] 18–24.9 kg/m^2^) and obese (BMI > 30 kg/m^2^) self-reported black and white South African women, who had been enrolled for previous studies and described in detail [18, 24]. Inclusion criteria included (1) age from 18 to 45 years; (2) no known diseases or taking medication for dyslipidemia, diabetes, hypertension, HIV/AIDS or any other metabolic disorders; and (3) not currently pregnant, lactating or postmenopausal. The study was approved by the Research Ethics Committee of the Faculty of Health Sciences of the University of Cape Town.

Basic anthropometric measurements, including height and weight, were taken. Body fat percentage was measured using dual-energy x-ray absorptiometry, while visceral and abdominal subcutaneous adipose tissue were measured using computed tomography as previously described [18]. Fasting serum samples were taken and stored at −80 °C prior to use.

### Biochemical measurements

#### HDL isolation

HDL was isolated from 200 μl aliquots of serum as follows: Serum samples were added to a mixture containing 1 part 500iu/ml heparin (Mucosal, Fresenius) and 2 parts 1.12 (mol/L) manganese chloride solution. Samples were centrifuged at 10 000 g for 1 h at 4 °C. The supernatant was dialysed against phosphate buffered saline (PBS, pH 7.4) in Spectra/Por 2 RC membrane (12 000–14 000 kDa) (GIC Scientific, 132676) and 200 μl aliquots were then dissolved in sodium bromide (275.5 mg/ml of supernatant), transferred to thick-wall polycarbonate ultracentrifuge tubes (Beckman, 343775) and centrifuged at 223 000 g for 20 h at 4 °C and the upper 70 μl layer was extracted. Purity was confirmed using 12.5 % reducing SDS-polyacrylamide gel electrophoresis (PAGE) stained with Coomassie Blue. The protein concentration of HDL was determined by the modified Lowry method [25]. All samples were analysed in duplicate.

#### Paraoxonase (PON) activity assay

Serum samples were diluted 1:10 in phosphate buffer containing 2 mmol/L CaCl_2_ (pH 8). Diluted serum was added to 96-well plates in triplicate and paraoxon-ethyl substrate (Sigma, D9286) was added. Absorbance at A_405_ was measured at 30 s intervals over 20 min. One Unit of activity is defined as 1 nmol of substrate hydrolysed per minute.

#### Platelet Activating Factor Acetylhydrolase (PAF-AH) activity assay

PAF-AH activity was measured in participant sera using the PAF Acetylhydrolase Assay Kit (Cayman Chemical, 760901). Briefly, serum was added to an equal volume of 5, 5′-dithio-*bis*-(2-nitrobenzoic acid) (DTNB; Ellman’s Reagent) and assay buffer in triplicate into clear 96-well plates. All wells were incubated with 2-thio PAF substrate and absorbance at A_412_ measured at 1 min time intervals for 20 min. One Unit of activity is defined as 1 μmol of substrate hydrolysed per minute.

#### Western blotting

Isolated HDL and serum samples from each of the participants were electrophoresed on reducing 12.5 % SDS-polyacrylamide (SDS-PAGE) gels with 1.5 μg of HDL protein or 8 ug of serum loaded per well. Samples were run over three separate gels with control samples repeated in each gel. Blots were transferred onto nitrocellulose membranes (Bio-Rad, 162-0113). Ponceau S staining was used to validate equal loading of wells. Blots were blocked in 5 % low fat milk powder in 0.05 % Tween in Tris-buffered Saline (TTBS, pH 7.5) and incubated overnight in primary mouse anti-PON-1 antibody (1:200) [26] and rabbit anti-PAF-AH (1:400) (Cayman Chemical, 160603). Blots were then washed in TTBS and incubated in goat anti-mouse-HRP conjugated secondary antibody (1:5000) (Bio-Rad, 170 6516) and goat anti-rabbit-HRP conjugated secondary antibody (1:2500) (Santa Cruz Biotechnology, sc-2313), respectively for 1 h at room temperature. Blots were thoroughly washed in TTBS prior to incubation in Amersham TM ECL^™^ Western blotting detection reagent (GE Healthcare, RPN2106). Blots were captured in the GeneGnome gel imager. Densitometry of PON-1 and PAF-AH blots was quantified using Quantity one software. PON-1 and PAF-AH relative expression data were corrected for control samples, repeated in each gel.

### Oxygen Radical Absorbance Capacity (ORAC) Assay

Isolated HDL samples were diluted (1:50) in phosphate buffer (pH 7.4) prior to analysis for the ORAC assay [27]. Briefly, a trolox standard curve of 0.078 nmol/L to 10 nmol/L was prepared. Fluorescein (3′, 6′ – dihydroxyspiro[isoberyofuran – 1[3H], 9′[9H] – xanthen] – 3-one) and AAPH (2,2′ – azobis (2-amidinopropane) dihydrochloride) were prepared fresh in phosphate buffer. The working fluorescein solution of fluorescein was 95.7 nmol/L and AAPH equated to 32.1 μmol/L per well. Fifty μl of trolox standards and HDL samples were added to wells in white 96-well plates (AEC-Amersham) along with AAPH and fluorescein. Fluorescence was measured over time using the Varian Cary Eclipse fluorescence spectrophotometer (Varian Australia Pty Ltd) (Excitation wavelength 485 nm, Emission wavelength 520 nm). Data were expressed as Trolox equivalents per volume.

#### Quantification of HDL anti-inflammatory function

Human umbilical vein endothelial cells (HUVEC) were purchased from Lonza and were cultured in T75 culture flasks according to supplier specifications. For experimental tests, 30 000 cells were seeded into 12-well culture plates and cultured in RPMI-1640 media supplemented with 20 % foetal calf serum (Biochrom BC/S0615), 1 ng/mL vascular endothelial growth factor (VEGF) (Sigma, V7259) and penicillin/streptomycin (Biowest, L0018). Five hours after seeding, the medium was changed and supplemented with HDL isolated according to optimised protocol at 10 μg/mL. Cells were treated with HDL overnight prior to stimulation with 20 ng/mL murine tumour necrosis factor alpha (TNF-α) (PeproTech, 315-01A) for 8 h. Cell pellets were harvested and stored in RNA Protect Cell Reagent (Qiagen, 76526) at −20 °C. RNA was isolated using the RNeasy Micro kit (Qiagen, 74004) and cDNA was synthesised using the High Capacity cDNA Reverse Transcriptase Kit (Life Technologies, 4368814). cDNA was quantified using the Qubit High Sensitivity RNA kit (Qiagen, Q32852) and Qubit Fluorometer (LifeTechnologies). cDNA was amplified for 25 cycles using the RT2 SYBR Green qPCR kit (Qiagen, 330500) in the RotorGene6000 (Corbit Lifesciences) with the following primers: VCAM-1 (sense), 5′-GAAGATGGTCGTGATCCTTG-3′ and (antisense), 5′-ACTTGACTGTGATCGGCTTC-3′. GAPDH (sense), 5′-CCACCCATGGCAAATTCCATGGCA-3′ and (antisense), 5′-TCTAGACGGCAGGTCAGGTCCACC-3′. Results indicate the mean of at least 3 independent experiments ± SEM.

#### Quantification of HDL subclass distribution

Serum HDL subclass was determined using the Lipoprint® HDL system (Quantimetrix, Redondo Beach, CA) [28]. Briefly, serum (25 μl) was mixed with Lipoprint loading gel (300 μl), containing Sudan black dye which binds proportionally to the cholesterol present in the sample. The mix was placed onto the upper part of the high resolution 3 % polyacrylamide gel. Photopolymerisation was carried out for 30 min at room temperature and electrophoresis was performed for 50 min at 3 mA per gel tube. After a rest period of 30 min, gel tubes were scanned and analysed using the Lipoware software. The VLDL and LDL remained at the origin [Retention Factor (Rf) = 0.0] while albumin migrated as the leading front (Rf = 1.0). Between these, 10 HDL bands could be detected. HDL-1, HDL-2 and HDL-3 were defined as large HDL; HDL-4, HDL-5, HDL-6 and HDL-7 were defined as intermediate HDL and HDL-8, HDL-9 and HDL-10 were defined as small HDL. Each subclass was quantified and expressed as a percentage of total HDL.

### Statistical analysis

Results are presented as mean ± standard error of mean (SEM). Two-way analysis of covariance, adjusting for age, was used to compare paraoxonase activity, PAF-AH activity, antioxidant capacity, relative VCAM expression and HDL subclass distribution between normal-weight and obese black and white women. Pearson correlation coefficients were used to explore the relationships between measures of serum lipids, body composition and HDL function and subclass.

## Results

The body composition, lipid profiles and additional physiological data of the participants have been previously published [18, 24] and summarised in Table 1. In brief, ethnic differences included lower visceral adipose tissue (VAT) and higher subcutaneous adipose tissue (SAT) in obese black women compared to obese white women. Critically, black women had lower HDL and total cholesterol concentrations than their white counterparts.

**Table 1:** Characteristics and serum lipids of participants included in the study

	White normal-weight (*n* = 12)	White obese (*n* = 9)	Black normal-weight (*n* = 8)	Black obese (*n* = 11)
Age (yr)	26 ± 2	34 ± 2^B, C^	23 ± 2	27 ± 2
BMI (kg/m^2^)	22.6 ± 0.7	33.4 ± 0.8^B, C^	22.8 ± 0.9	38.5 ± 0.7^D^
Fat (kg)	19.2 ± 1.4	40.4 ± 1.6^C^	17.1 ± 1.7	44.9 ± 1.5^D^
Body fat (%)	29.5 ± 1.4	43.7 ± 1.6^C^	30.0 ± 1.7	46.7 ± 1.5^D^
VAT area (cm^2^)	62.2 ± 11.3	144.9 ± 13.1^B, C^	56.9 ± 13.9	95.6 ± 11.8^D^
SAT area (cm^2^)	187.0 ± 19.1	471.6 ± 22.0^B, C^	175.2 ± 23.4	594.2 ± 19.9^d^
Serum lipids				
HDL cholesterol (mmol/L)	1.7 ± 0.1^a^	1.5 ± 0.1^B^	1.3 ± 0.1	1.0 ± 0.1
LDL cholesterol (mmol/L)	2.0 ± 0.2	2.5 ± 0.2	2.1 ± 0.2	2.1 ± 0.2
Triglycerides (mmol/L)	0.9 ± 0.1	1.0 ± 0.1	0.5 ± 0.1	0.8 ± 0.1
Total cholesterol (mmol/L)	4.1 ± 0.2	4.6 ± 0.3^B^	3.6 ± 0.3	3.5 ± 0.2

Values are unadjusted means ± SEM. All *p* values are adjusted for age

^a^ *p* < 0.05 white normal-weight vs black normal-weight

^B^ *p* < 0.01 white obese vs black obese

^C^ *p* < 0.01 normal-weight vs obese white

^d^ *p* < 0.05 and ^D^ *p* < 0.01 normal-weight vs obese black

### PON activity in normal-weight and obese black and white women

**Fig. 1:**
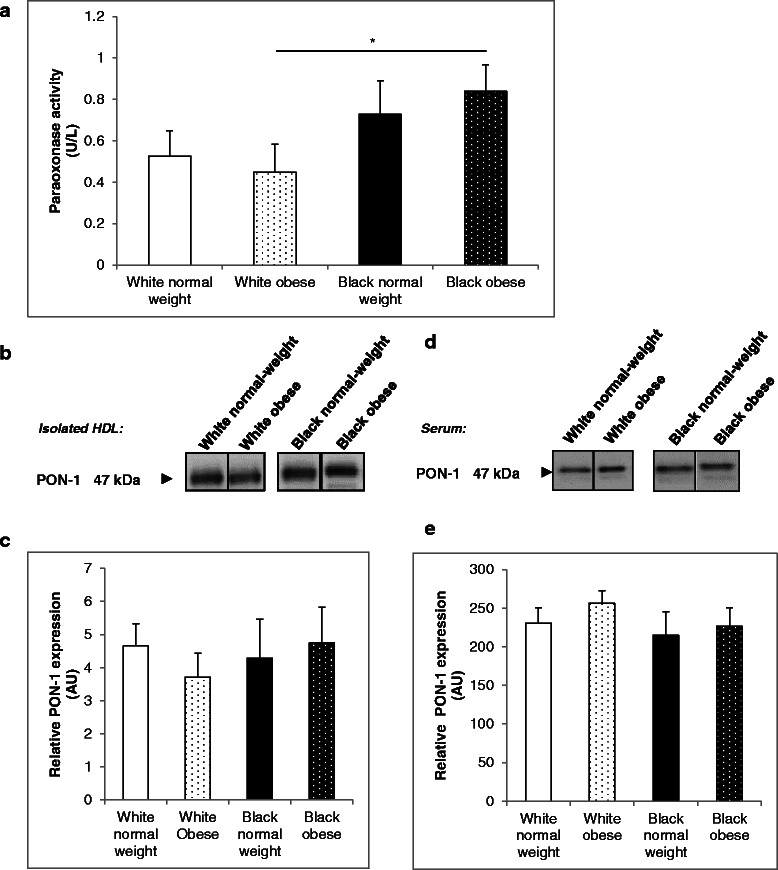
Paraoxonase activity and protein expression in white and black women. Paraxonase activity of diluted sera was measured at A_405_ over a 20 min time interval using the paraoxon-ethyl substrate. One unit of activity is defined as 1 nmol of substrate disintegrated per minute (**a**). Isolated HDL (**b**–**c**) and participant sera (**d**–**e**) were run on reducing 12.5 % SDS-PAGE gels and transferred to nitrocellulose membrane. Ponceau S staining was used to confirm equal loading. Blots were probed with mouse anti-PON-1 antifbody. Results are representative of randomized experiments (**b**) and (**d**). Densitometry of PON-1 expression in HDL (**c**) and sera (**e**) Results represent means ± SEM * *p* < 0.05

PON activity of normal-weight and obese, black and white women is presented in Fig. 1a. Black women had significantly higher PON activity levels than white women (0.78 ± 0.10 U/L vs 0.49 ± 0.09 U/L, *p* < 0.05), with the effect being more pronounced in obese black women compared to obese white women (0.84 ± 0.13 U/L vs 0.45 ± 0.14 U/L, *p* < 0.05) (Fig. 1a). Irrespective of ethnicity, PON activity did not differ subject to obesity.

In order to explore whether differences in PON activity levels were simply due to differences in PON-1 protein expression in HDL, Western blotting was performed on isolated HDL and serum. There were no significant differences in HDL-associated PON-1 protein levels between black and white women, nor between normal-weight and obese women (Fig. 1b and c). Similarly, there were no differences in PON-1 serum expression between black and white women, nor between normal-weight and obese women (Fig. 1d and e). Full blot pictures are shown in Additional file 1: Figure S1.

Table 2 shows that PON activity correlated positively with LDL levels in both black and white women (*p* < 0.05), positively with total cholesterol (*p* < 0.005) in black women and negatively with total HDL (*p* < 0.005) in white women.

**Table 2:** Associations between HDL functionality measures, HDL subclass, body composition and serum lipids in black and white South African women

	Ethnicity	Age (year)	BMI (kg/m^2^)	Fat (kg)	VAT (cm^2^)	Total SAT (cm^2^)	HDL (mmol/L)	LDL (mmol/L)	Triglycerides (mmol/L)	Total cholesterol (mmol/L)
PON Activity	Black	*r* = −0.07	*r* = 0.30	*r* = 0.26	*r* = 0.19	*r* = 0.19	*r* = 0.29	*r* = 0.59*	*r* = 0.13	*r* = 0.66**
	White	*r* = −0.40	*r* = 0.14	*r* = 0.16	*r* = −0.25	*r* = −0.09	*r* = −0.62***	*r* = 0.45*	*r* = 0.21	*r* = 0.28
PAF-AH activity	Black	*r* = −0.18	*r* = −0.44	*r* = −0.47*	*r* = −0.25	*r* = −0.48*	*r* = 0.05	*r* = 0.65***	*r* = −0.51*	*r* = 0.53*
	White	*r* = 0.02	*r* = 0.42	*r* = 0.39	*r* = −0.02	*r* = −0.10	*r* = −0.37	*r* = 0.85***	*r* = 0.22	*r* = 0.80***
Large HDL subclass	Black	*r* = −0.52*	*r* = −0.32	*r* = −0.30	*r* = −0.18	*r* = −0.31	*r* = 0.69**	*r* = 0.067	*r* = −0.19	*r* = 0.25
	White	*r* = −0.31	*r* = −0.52*	*r* = −0.48*	*r* = −0.48*	*r* = −0.48*	*r* = 0.32	*r* = −0.49*	*r* = −0.48*	*r* = −0.48*
Intermediate HDL subclass	Black	*r* = 0.24	*r* = 0.43	*r* = 0.41	*r* = 0.17	*r* = 0.45	*r* = −0.64***	*r* = 0.17	*r* = −0.15	*r* = −0.11
	White	*r* = 0.40	*r* = 0.46*	*r* = 0.44*	*r* = 0.58*	*r* = 0.51*	*r* = −0.33	*r* = 0.36	*r* = 0.52*	*r* = 0.34
Small HDL subclass	Black	*r* = 0.52*	*r* = 0.08	*r* = 0.08	*r* = 0.11	*r* = 0.06	*r* = −0.29	*r* = −0.24	*r* = 0.42	*r* = −0.25
	White	*r* = 0.20	*r* = 0.52*	*r* = 0.47*	*r* = 0.34	*r* = 0.41	*r* = −0.27	*r* = 0.55**	*r* = 0.40	*r* = 0.55**

Values are Pearson correlation coefficients

*BMI* body mass index, *VAT* visceral adipose tissue, *SAT* subcutaneous adipose tissue

* *p* < 0.05, ** *p* < 0.01, *** *p* < 0.005

### PAF-AH activity in normal-weight and obese black and white women

**Fig. 2:**
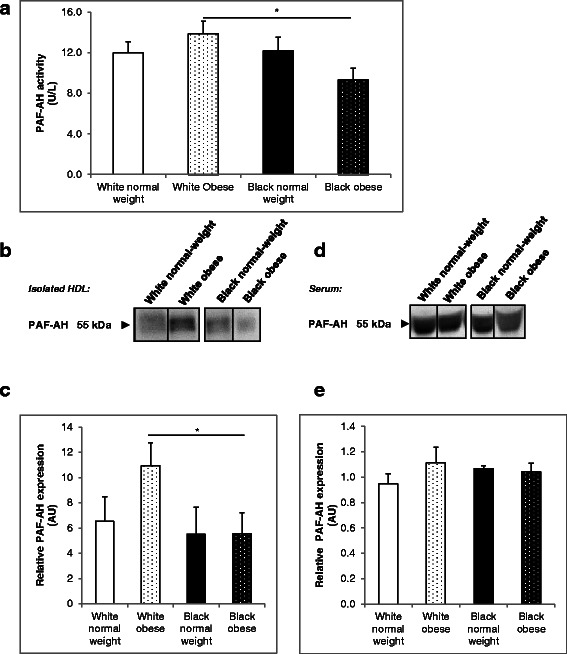
PAF-AH activity and protein expression in white and black women. PAF-AH activity of diluted sera was measured at A_412_ over a 20 min time interval using the PAF Acetylhydrolase Assay Kit. One unit of activity is defined as 1 μmol of substrate disintegrated per minute (**a**). Isolated HDL (**b**–**c**) and participant sera (**d**–**e**) were run on reducing 12.5 % SDS-PAGE gels and transferred to nitrocellulose membrane. Ponceau S staining was used to confirm equal loading. Blots were probed with rabbit anti-PAF-AH antibody. Results are representative of randomized experiments (**b**) and (**d**). Densitometry of PAF-AH expression in HDL (c) and sera (**e**) Results represent means ± SEM * *p* < 0.05

There were no differences in PAF-AH activity between black and white women, however, obese black women had significantly lower PAF-AH activity levels than obese white women (9.34 ± 1.15 U/L vs 13.89 ± 1.21 U/L, *p* < 0.05) (Fig. 2a). PAF-AH activity did not differ subject to obesity.

When examining PAF-AH protein expression in isolated HDL, we found that obese black women had significantly lower levels of HDL-associated PAF-AH than obese white women (5.5 ± 1.7 vs 10.9 ± 1.8 Arbitrary units, AU, *p* < 0.05) (Fig. 2b and c), which corresponds to lower PAF-AH activity (*r* = 0.54, *p* < 0.005). However, there were no differences between black and white obese and normal-weight women in PAF-AH serum expression (Fig. 2d and e). Full blots are shown in Additional file 1: Figure S2.

PAF-AH was positively correlated with LDL (*p* < 0.005) and total cholesterol (*p* < 0.005) concentrations in both black and white women (Table 2). In black women only, increased PAF-AH activity was associated with reduced fat mass, SAT and triglyceride concentrations (*p* < 0.05).

### Antioxidant capacity and anti-inflammatory function of isolated HDL

No obesity-related or ethnic differences were observed for the anti-inflammatory function and antioxidant capacity of isolated HDL (Additional file 1: Figures S3 and S4).

### HDL subclass distribution in normal-weight and obese black and white women

**Fig. 3:**
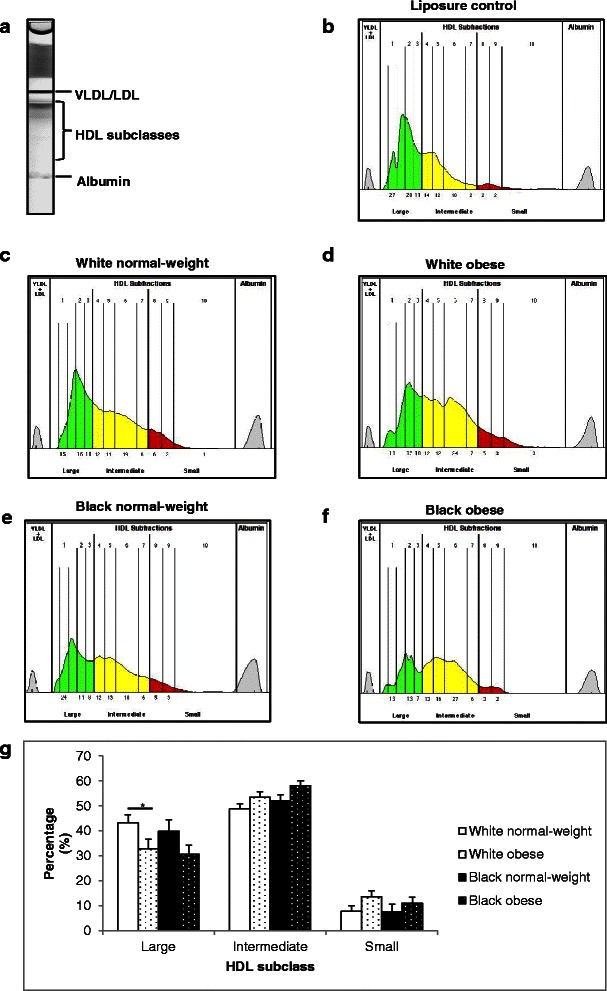
Distribution of HDL subclasses in participant sera. Subject sera was analysed using the Lipoprint® system and analysed using Lipoware software. Representative scan (**a**) and scan result (**b**) of Liposure control indicating HDL subclass bands. Representative scan results from white and black obese and normal-weight women (**c**–**f**). Percentages of large, intermediate and small HDL subclasses (**g**). VLDL – Very low density lipoprotein. Results represent means ± SEM. * *p* < 0.05

Figure 3 shows the distribution of large, intermediate and small HDL subclasses, quantified using the Lipoprint® system. Scans (Fig. 3a) were quantified, producing unique HDL subclass profiles (Fig. 3b–f). HDL subclass distribution was different between obese and normal-weight women with less large HDL (−10 %, *p* < 0.05) and significantly more intermediate HDL (+6 %, *p* < 0.05) in the obese compared to the normal-weight women (Fig. 3g), which was largely driven by differences between the normal-weight and obese white women (43.1 ± 3.4 % vs 32.8 ± 3.8 % for large HDL, *p* < 0.05) (Fig. 3g). There were no differences in the percentages of small HDL subclasses between groups.

In the white women, the greater proportion of large HDL subclasses was associated with higher BMI, fat mass, VAT and SAT, as well as higher LDL and triglyceride concentrations (*p* < 0.05, Table 2). In black women, the greater proportion of large HDL was associated with a younger age (*p* < 0.05) and higher HDL-C (*p* < 0.005). Conversely, a greater proportion of intermediate HDL was associated with lower BMI, fat mass, VAT and SAT in white women. A greater proportion of small HDL was associated with increased BMI and fat mass in white women.

## Discussion

In this preliminary study we aimed to examine whether ethnicity and obesity may be associated with differences in HDL functionality and subclass distribution. Our data showed that, despite lower levels of HDL-C, black women had higher PON activity levels compared to white women. In contrast, the activity and protein expression of PAF-AH was lower in obese black compared to obese white women. Obesity was not associated with a difference in the activity of these enzymes, but was associated with a shift in HDL subclass from large to intermediate and small HDL, an effect which was largely driven by differences between normal-weight and obese white women.

The relatively low incidence of myocardial infarction in the South African black population has long been thought to be related to an increase in HDL-C levels in comparison to white populations. However, recent studies provide evidence for lower HDL-C in South African black populations compared South African white populations, despite clear reduced risk of acute myocardial infarction [6, 18]. Recent research suggests that the quality of HDL, rather than its quantity, may be a more important factor to consider as a cardiovascular risk factor (see review, [23]).

Low serum PON activity levels have been associated with an increased risk for major adverse cardiovascular events (MACE), including myocardial infarction and stroke in an American study [29]. The novel finding of higher PON activity levels in black women compared to white women, independent of protein expression levels is in contrast to other studies in the USA that found that African Americans had lower or similar PON activity levels than their white counterparts, despite similar HDL-C concentrations [30, 31].

Accordingly, we hypothesise that the low rate of cholesterol-attributable mortality [15], and particularly the low incidence of myocardial infarction in black populations [6] may be explained by higher PON activity levels. However, this hypothesis would need to be tested, as a study in a mixed race South African population found that PON activity was not an accurate predictor of cardiovascular risk [32].

Our data also indicated that increased PON activity in black women was not related to a greater amount of HDL-associated PON-1 protein, which may relate to genetic factors. PON-1 polymorphisms, such as the promotor region small nucleotide polymorphism (SNP) L-55 M, causes a reduction in serum PON activity and is much less frequent in oriental and black populations [33, 34]. Additionally, PON-1 polymorphisms can result in lower activity with PON-1 serum protein levels unchanged [34].

We showed associations between increased PON activity and typical markers of risk, including lower LDL and triglyceride concentrations, which have been reported previously in an American white young adult population [35]. Of interest was a negative correlation between PON activity and HDL-C in white but not black women. Failure to show this association in black women is unexpected because HDL is associated with PON and has been shown in other studies to be negatively correlated with HDL-C [36, 37]. As PON activity remains consistently high in black women, it is possible that PON activity may be a marker of HDL function in black women, independent of HDL-C levels.

PAF-AH is another HDL-associated enzyme whose primary physiological role is maintenance of PAF metabolism and anti-thrombotic functions [38, 39]. Our data show significantly lower PAF-AH activity in obese black women compared to obese white women. Overall, PAF-AH activity was correlated with PAF-AH expression in HDL while PAF-AH serum expression remained unchanged amongst the groups. Obese black women therefore expressed significantly less HDL-associated PAF-AH than obese white women. Furthermore, reduced PAF-AH activity was associated with increased fat mass and SAT in black women only. PAF-AH is mainly associated with LDL and a smaller proportion with HDL, which translate into different physiological functions of PAF-AH activity [40]. Reduced HDL-associated PAF-AH activity has been shown to be associated with increased risk of cardiovascular disease [41]. Accordingly, our data suggest that reduced HDL-associated PAF-AH activity in obese black women may be associated with a reduction in anti-atherogenic HDL function. However, previous studies have shown that PON activity modulates HDL-associated PAF-AH activity [41]. We, therefore, propose that higher PON activity in obese black women may circumvent reductions in PAF-AH activity. However, further studies are needed to test this hypothesis.

Surprisingly, despite the findings regarding PON activity, we did not find any significant between group differences in antioxidant capacity of isolated HDL. This was similarly found in a study comparing diet induced weight loss in overweight American participants [42]. We propose that, in the ORAC assay, measurement of total antioxidant capacity may produce different results to a specific antioxidant assay such as PON activity, owing to contributions of additional HDL components. Similarly no differences in the expression of VCAM in HUVEC cells treated with isolated HDL were found. Since HDL has been shown to reduce expression of a number of endothelial adhesion molecules, additional markers would need to be used to further confirm the findings in this study.

To our knowledge, this is the first report of the distribution of HDL subclasses in an African population. In our study a similar profile of HDL subclass distribution was observed between black and white women. However, obesity was associated with reduced large HDL subclasses and concomitant higher intermediate and small subclasses; largely driven by differences between normal-weight and obese white women. In support of this finding, markers of increased adiposity, BMI, fat mass, percentage fat, VAT and SAT correlated with differences in large and intermediate subclasses in white but not black women. This shift has been previously shown in non-African male and female obese populations, where a decrease in average HDL particle size and increased concentrations of smaller HDL subclasses were reported [43–45]. In this case, HDL subclass distribution does not explain changes in functionality related to ethnicity. HDL subclasses were not significantly different between normal-weight and obese black women, while HDL subclasses differed by obesity in white women. A similar trend was observed in LDL subclasses in the same population of women, where differences in LDL subclass in normal-weight and obese women were observed in white but not black women [18].

Longitudinal data on a black South African cohort also indicate that despite increases in weight, HDL-C concentrations remained consistently low in black women [46]. In spite of this, we show that black women display improved HDL antioxidant functionality in comparison to white women, indicating the importance in measurement of HDL quality instead of total HDL cholesterol levels.

## Conclusions

We acknowledge that the small sample size limits the conclusions which can be drawn, however our data indicate that obesity and ethnicity affect HDL functionality and HDL subclass. We, therefore, suggest that future studies examining the association between HDL and cardiovascular risk should focus on examining the role of HDL subclass and functionality. We considered a number of functionality assays in this study, however, additional measures such as reverse cholesterol efflux can be considered in similar studies in the future. Longitudinal studies are required to determine if HDL subclass and function are indeed important risk factors for cardiovascular disease.

### Ethics approval

The study was approved by the Research Ethics Committee of the Faculty of Health Sciences of the University of Cape Town, reference 053/2003.

### Consent for publication

Not applicable.

## Additional file

Additional file 1: Figure S1. Paraoxonase protein expression in white and black women. Isolated HDL (a–c) and serum (d–f) from each participant was randomly loaded and run on reducing 12.5 % SDS-PAGE gels and transferred to nitrocellulose membrane. Ponceau S staining was used to confirm equal loading. Blots were probed with mouse anti-PON-1 antibody. WN = White normal-weight. WO = White obese. BN = Black normal weight. BO = Black obese. **Figure S2.** PAF-AH protein expression in white and black women. Isolated HDL (a–c) and serum (d–f) from each participant was randomly loaded and run on reducing 12.5 % SDS-PAGE gels and transferred to nitrocellulose membrane. Ponceau S staining was used to confirm equal loading. Blots were probed with rabbit anti-PAF-AH antibody. WN = White normal-weight. WO = White obese. BN = Black normal weight. BO = Black obese. **Figure S3.** Vascular Cell Adhesion Molecule (VCAM) expression in endothelial cells treated with HDL. HUVEC cells were treated overnight with 10 μg/ml subject HDL. Cells were exposed to 20 ng/ml tumour necrosis factor (TNF) for 8 h. Cell lysates were harvested and stored in RNAprotect reagent prior to RNA extraction, followed by cDNA synthesis and quantitative real time PCR. Results are presented relative to a no-HDL treatment control. Results are means of 3 independent experiments ± SEM. **Figure S4.** Antioxidant capacity of isolated HDL. Isolated subject HDL was diluted in phosphate buffer and measured using the Oxygen Radical Absorbance Capacity (ORAC) assay. (PDF 403 kb)

## Abbreviations

BMI: body mass index
DTNB: 5, 5'-dithio-bis-(2-nitrobenzoic acid)
HDL-C: high-density lipoprotein-cholesterol
HUVEC: human umbilical vein endothelial cells
LDL: low-density lipoprotein
PAF-AH: platelet activating factor acetylhydrolase
PON: paraoxonase
SAT: subcutaneous adipose tissue
SDS-PAGE: SDS-polyacrylamide
TNF- α: tumour necrosis factor alpha
VAT: visceral adipose tissue
VCAM: vascular cell adhesion molecule
VEGF: vascular endothelial growth factor
