# Supplementary material for: Association between ethnicity and obesity with high-density lipoprotein (HDL) function and subclass distribution
Source: Lipids Health Dis. 2016 May 11;15:92. doi: 10.1186/s12944-016-0257-9 (PMC4866302; doi:10.1186/s12944-016-0257-9)
Supplement: Additional file 1: Figure S1. — Paraoxonase protein expression in white and black women. Isolated HDL (a–c) and serum (d–f) from each participant was randomly loaded and run on reducing 12.5 % SDS-PAGE gels and transferred to nitrocellulose membrane. Ponceau S staining was used to confirm equal loading. Blots were probed with mouse anti-PON-1 antibody. WN = White normal-weight. WO = White obese. BN = Black normal weight. BO = Black obese. Figure S2. PAF-AH protein expression in white and black women. Isolated HDL (a–c) and serum (d–f) from each participant was randomly loaded and run on reducing 12.5 % SDS-PAGE gels and transferred to nitrocellulose membrane. Ponceau S staining was used to confirm equal loading. Blots were probed with rabbit anti-PAF-AH antibody. WN = White normal-weight. WO = White obese. BN = Black normal weight. BO = Black obese. Figure S3. Vascular Cell Adhesion Molecule (VCAM) expression in endothelial cells treated with HDL. HUVEC cells were treated overnight with 10 μg/ml subject HDL. Cells were exposed to 20 ng/ml tumour necrosis factor (TNF) for 8 h. Cell lysates were harvested and stored in RNAprotect reagent prior to RNA extraction, followed by cDNA synthesis and quantitative real time PCR. Results are presented relative to a no-HDL treatment control. Results are means of 3 independent experiments ± SEM. Figure S4. Antioxidant capacity of isolated HDL. Isolated subject HDL was diluted in phosphate buffer and measured using the Oxygen Radical Absorbance Capacity (ORAC) assay. (PDF 403 kb) [file 12944_2016_257_MOESM1_ESM.pdf]

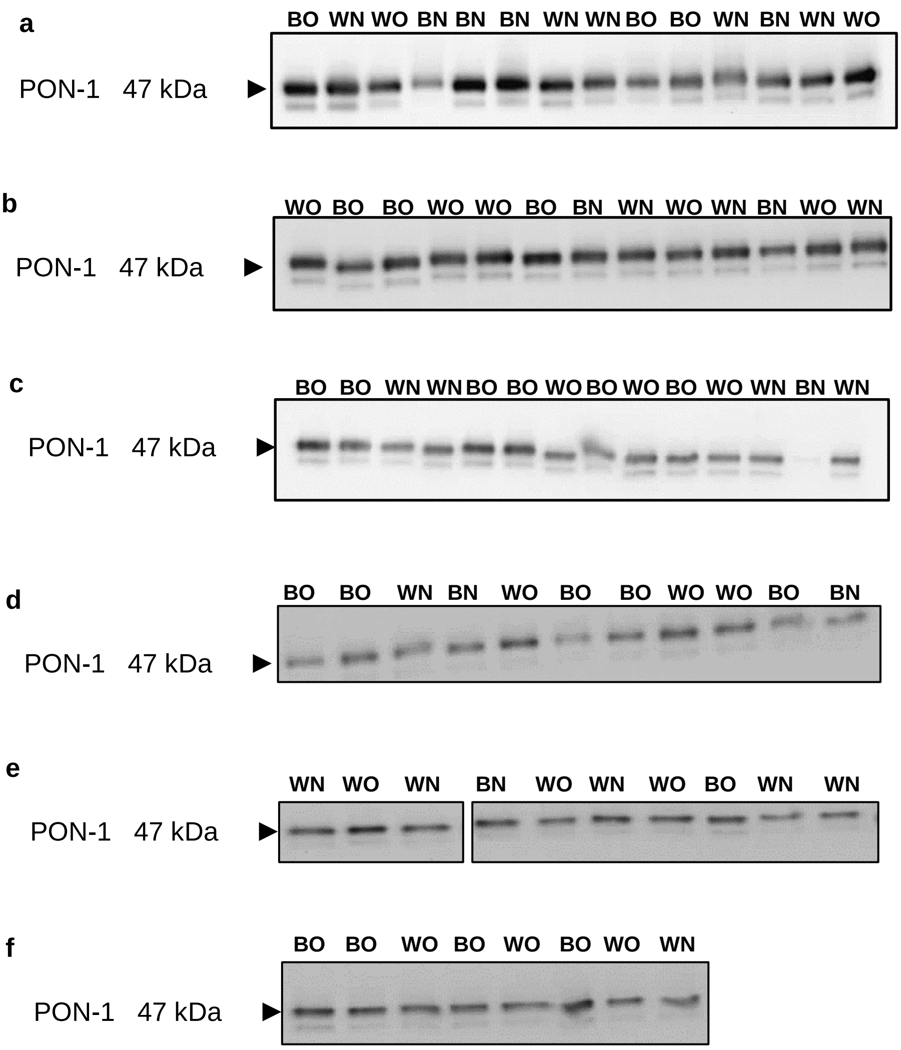


**Supplementary Fig 1. Paraoxonase protein expression in white and black women.** Isolated HDL (a-c) and serum (d-f) from each participant was randomly loaded and run on reducing 12.5% SDS-PAGE gels and transferred to nitrocellulose membrane. Ponceau S staining was used to confirm equal loading. Blots were probed with mouse anti-PON-1 antibody. WN = White normal-weight. WO = White obese. BN = Black normal weight. BO = Black obese.

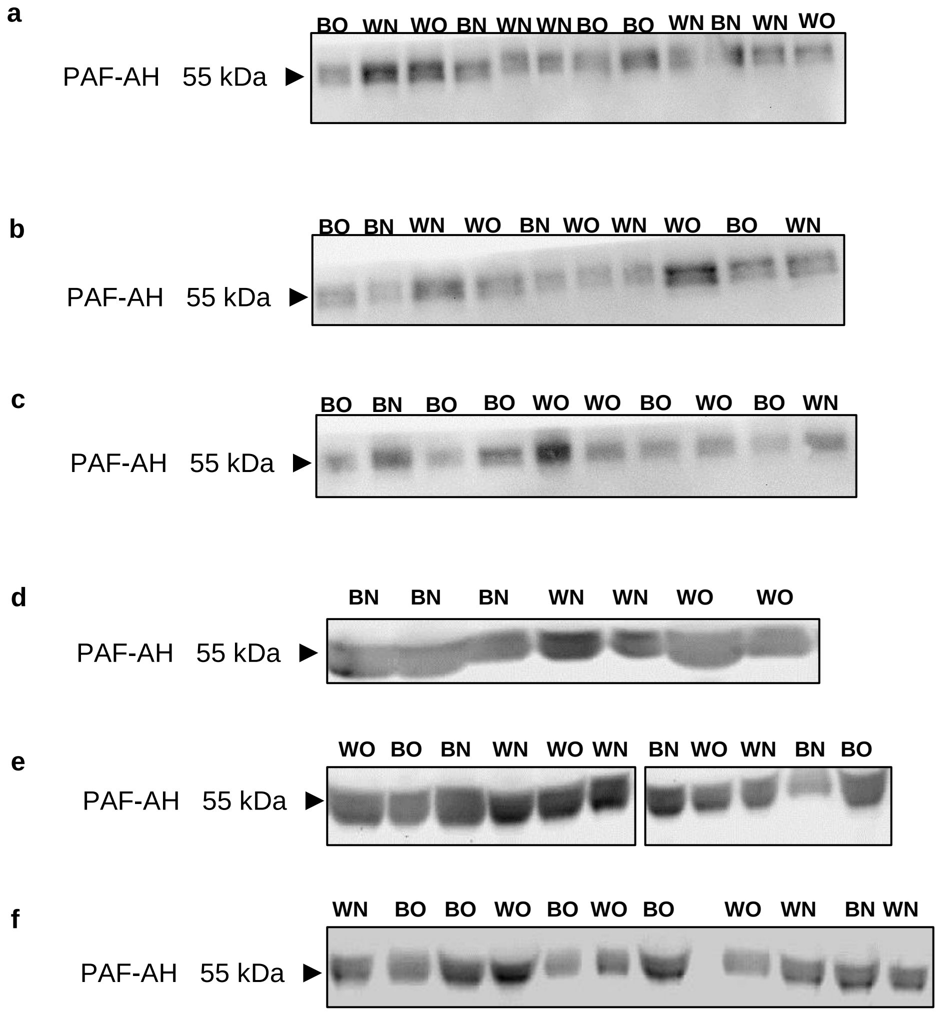


**Supplementary Fig 2. PAF-AH protein expression in white and black women.** Isolated HDL (a-c) and serum (d-f) from each participant was randomly loaded and run on reducing 12.5% SDS-PAGE gels and transferred to nitrocellulose membrane. Ponceau S staining was used to confirm equal loading. Blots were probed with rabbit anti-PAF-AH antibody. WN = White normal-weight. WO = White obese. BN = Black normal weight. BO = Black obese.

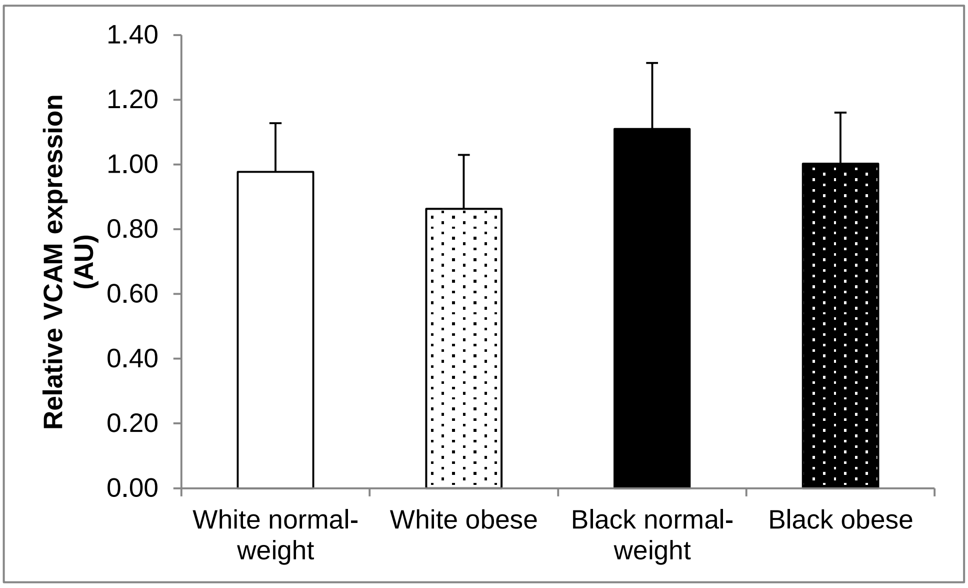


**Supplementary Fig 3. Vascular Cell Adhesion Molecule (VCAM) expression in endothelial cells treated with HDL.** HUVEC cells were treated overnight with 10 µg/ml subject HDL. Cells were exposed to 20 ng/ml tumour necrosis factor (TNF) for 8 hours. Cell lysates were harvested and stored in RNAprotect reagent prior to RNA extraction, followed by cDNA synthesis and quantitative real time PCR. Results are presented relative to a no-HDL treatment control. Results are means of 3 independent experiments ± SEM.

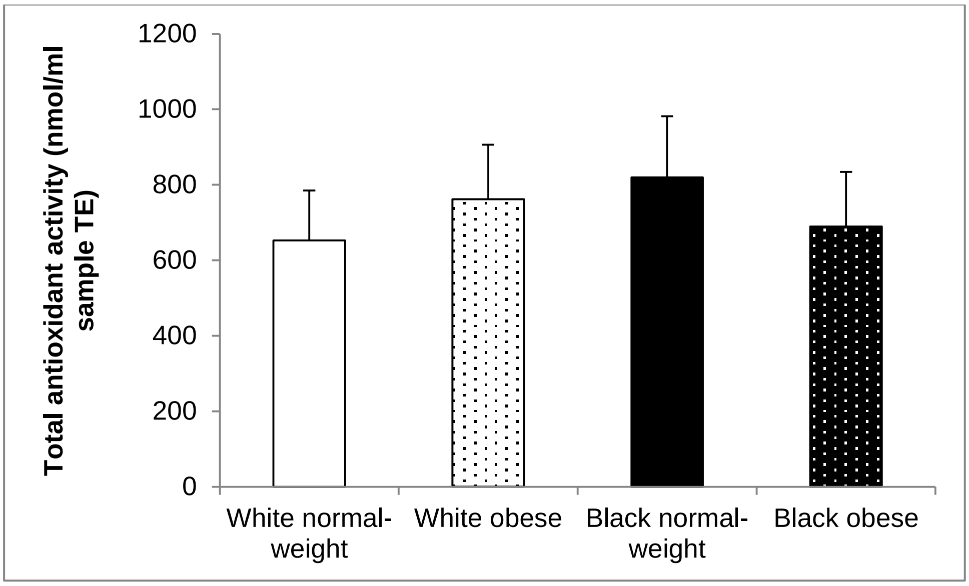


**Supplementary Fig 4. Antioxidant capacity of isolated HDL.** Isolated subject HDL was diluted in phosphate buffer and measured using the Oxygen Radical Absorbance Capacity (ORAC) assay.
